# Correction: Attacking quantum key distribution by light injection via ventilation openingss

**DOI:** 10.1371/journal.pone.0244010

**Published:** 2020-12-09

**Authors:** Juan Carlos Garcia-Escartin, Shihan Sajeed, Vadim Makarov

There is an error in affiliations for the third author. Vadim Makarov is not affiliated with #4
