# Supplementary figures and images for: Attacking quantum key distribution by light injection via ventilation openings
Source: PLoS One. 2020 Aug 3;15(8):e0236630. doi: 10.1371/journal.pone.0236630 (PMC7398518; doi:10.1371/journal.pone.0236630)

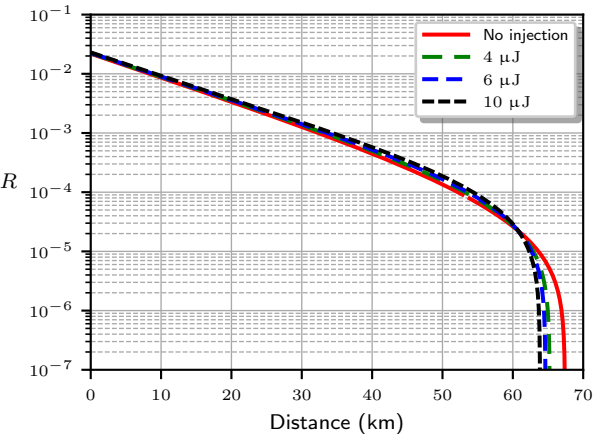

Supplement: S1 Data — (ZIP) [file pone.0236630.s001.zip › SupportingData/Clavis/RBB84.pdf]

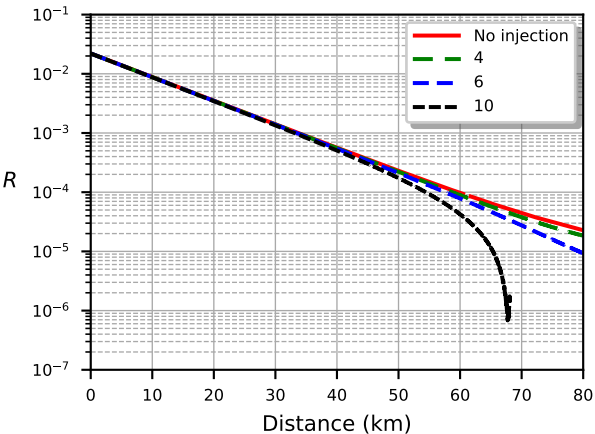

Supplement: S1 Data — (ZIP) [file pone.0236630.s001.zip › SupportingData/Clavis/RBB84Opt.pdf]

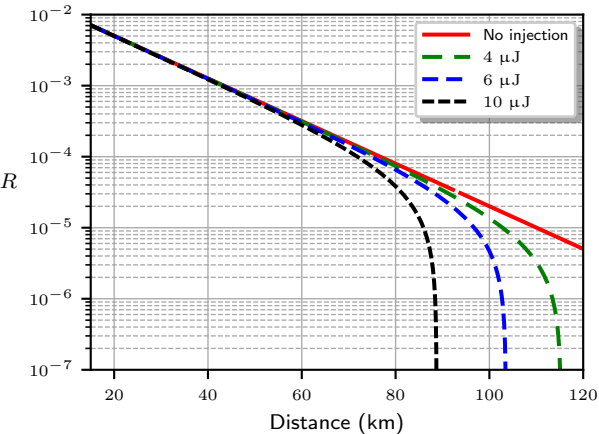

Supplement: S1 Data — (ZIP) [file pone.0236630.s001.zip › SupportingData/Clavis/RSARG04.pdf]
