# Supplementary material for: Attacking quantum key distribution by light injection via ventilation openings
Source: PLoS One. 2020 Aug 3;15(8):e0236630. doi: 10.1371/journal.pone.0236630 (PMC7398518; doi:10.1371/journal.pone.0236630)
Supplement: Attachment — Submitted filename: report-injctAttcks.pdf [file pone.0236630.s002.pdf]

The manuscript by Juan Carlos Garcia-Escartin *et al.* presents an information leakage attack performed on quantum key distribution (QKD) and quantum random number generator (QRNG) devices. One of the main assumptions when it comes to discussing device-dependent security is that the adversary 'Eve' does not have any physical access to them. However, forbidding access from an electromagnetic point of view is not at all easy, and the plethora of TEMPEST and Trojan attacks in the last decades are a testimony to that.

The attack here hinges on a line of sight (LOS) that could be unwittingly offered to Eve via the ventilation openings in the device: in an otherwise secure and physically inadmissible (to Eve) lab/office, a device placed e.g. next to a wall with its ventilation holes facing a window could be injected with optical radiation by Eve from a building across the street. Specifically, as per the proof of concept demonstration here, Eve could then manipulate the mean photon number of the quantum states modulated by the QKD transmitter and bias the output of the QRNG in a controlled manner. If the device operator does not notice such an ongoing attack, the secret key / random number sequence from the QKD / QRNG device could be compromised.

The manuscript is well written: equations are in an easy-to-read format, sectioning is logical, and photos, schematics, and graphs nicely illustrate the experiment. While the exploited vulnerability reported here is definitely novel and ingenious, the seriousness of its impact on the security of the system is somewhat debatable. Based on experimental measurements conducted on (a fiber spool inside) the QKD transmitter and heuristics, the authors conclude that Eve would have to shine a **few 100s of Watts of laser light** to be able to inject a sufficient number of photons (on average) into the transmitter that then cause security violations. Would such a strong beam of light have absolutely no other influence? Would it not *fry* for instance the plastic sheath covering the fiber spool? Or badly injure a poor device operator who might accidentally walk into the beam path[1]? Or could multiple reflections and scatterings from such strong light beam result in some abnormal functioning of the device, thus disclosing the attack?

What I mean to say is, there are likely to be several unintended effects an actual attack may bring, and this has not been addressed at all. This group has in the past specialized in a variety of optical damage attacks so ideally speaking, they should perform some more measurements with the highest possible powers available and then include a frank discussion about telltales that Eve might leave behind. Also, given that the injection efficiency (free-space to fiber coupling via the reversible loss mechanisms) seems to be < -120 dB, it might make sense to compare the effectiveness of this attack with a traditional trojan attack where Eve shines light (through the quantum channel) at a wavelength long enough to prevent exciting the classical detectors photoelectrically. And they should definitely add a citation to a telecom wavelength source that produces 'realistically achievable c.w. power in the range of 200 – 500 W'.

Some other questions and comments:

1. I guess the reason for using c.w. light for the demonstration here was to avoid the problem of synchronization of optical pulses to the gates of the single photon detector. But for an actual attack, isn't Eve somewhat limited by using c.w. light? As in, would a pulsed laser not be more practical?

2. Section B, Page 8: "Comparing the coupling of light at 1310 and 1550 nm, we see that more photons enter the fiber at the lower 1310 nm wavelength, where Rayleigh scattering is stronger for silica fibers."
   A natural question that arises here is: if more photons enter at 1310 nm, why is that not chosen as the attack wavelength? In general, what were the beam waists for these 2 wavelengths and how does the coupling vary if the beam waist is changed?

3. Section B, Page 14: "We assume Eve knows the phase of the injected photons before the modulation and that she can establish their phase difference with respect to the reference pulses from Bob."
   Might it not be simpler for Eve to discriminate the phase using homodyne detection, as was done in NJP 16, 123030 (2014)? (A phase reference can also be easily established since time periods with no modulation are equivalent to when a phase of 0 is modulated).

4. Section B, Page 15: "Eve manages to inject a pulse with mean photon number μ_{inj} towards the PM direction."
   It would be nice to elaborate this sentence to avoid confusion. If I understood correctly, what is actually

[1]The probability of a human operator walking into the beam path is probably a few orders of magnitude higher than the probability of Eve getting a LOS through ventilation holes to launch such an attack in the first place.

happening is that Eve shines a bright pulse which, due to the reversible loss coupling mechanism, results in a pulse with mean photon number μ_{inj} that travels in the fiber towards the PM.

5. Section C, Page 16: Was the fluctuation around the $N = 60.8$ value in the count rate negligible? If not, then mention that as well.

6. Section C, Equation 8: Add expression with an integer exponent to the RHS (e.g., $\mu_{ext} = 3.46 \times 10^{-6.3} = 1.73 \times 10^{-6}$) because otherwise it may cause a potential confusion when compared with μ_{inj}.

7. The caption in Fig. 6 reads "we take advantage of a ventilation hole at the top of the box". Is this is the beam splitter box? And it might make it easier for the reader to understand by adding the blue ray path in Fig. 6(b) as well.
