# Supplementary material for: Attacking quantum key distribution by light injection via ventilation openings
Source: PLoS One. 2020 Aug 3;15(8):e0236630. doi: 10.1371/journal.pone.0236630 (PMC7398518; doi:10.1371/journal.pone.0236630)
Supplement: Attachment — Submitted filename: response.pdf [file pone.0236630.s003.pdf]

REVISED VERSION

Dear Editors,

We send for your consideration a revised version of our paper "Attacking quantum key distribution by light injection via ventilation openings". We have taken into account the comments from the reviewers and have modified the paper accordingly.

First, we give a general description of the changes and later a detailed response to the reviewers. The most relevant changes are:

- Section III has been revised to improve the description of the experimental requirements for our attack. In particular:
  - Section III.B now discusses the properties of the incoming laser light (beam and time shape) and how they relate to the attack efficiency and the required laser power.
  - Section III.D has been reformulated and, instead of talking about the laser powers that allow for a successful attack, we talk about the required energy that an attacker must send during each QKD time bin in which the modulator is active (20 ns). This is more natural when speaking about pulsed lasers and helps to study the feasibility of the attack in terms of the energy per pulse.
  - Apart from modifying the figures, the discussion at the end of the Section has been expanded and gives the parameter range for which a pulsed laser can compromise the key exchange. We give a list of commercial lasers used for LIDAR applications which could be adapted to this purpose.
- In Section IV we have modified the Figure with the picture of the QRNG to make clear its correspondence with the block diagram. We have also expanded the discussion on the effects of the injected light on the bit statistics and commented how the sequences we recorded under the attack fail different randomness tests. We provide the sample sequences in the Supporting Data.
- We have added the requested Supporting Data with details about the experiments. In particular, we include:
  - Source code from which we generated the key rate graphs.
  - Experimental data for the QKD experiment with a description of the equipment we used to collect it.
  - Samples from the QRNG including reports with the randomness test results from the `ent` and `rngtest` Linux utilities.

With these changes we hope we have answered all the concerns of the reviewers.

A. Answers to reviewer 1:

The manuscript by Juan Carlos Garcia-Escartin et al. presents an information leakage attack performed on quantum key distribution (QKD) and quantum random number generator (QRNG) devices. One of the main assumptions when it comes to discussing device-dependent security is that the adversary 'Eve' does not have any physical access to them. However, forbidding access from an electromagnetic point of view is not at all easy, and the plethora of TEMPEST and Trojan attacks in the last decades are a testimony to that. The attack here hinges on a line of sight (LOS) that could be unwittingly offered to Eve via the ventilation openings in the device: in an otherwise secure and physically inadmissible (to Eve) lab/office, a device placed e.g. next to a wall with its ventilation holes facing a window could be injected with optical radiation by Eve from a building across the street. Specifically, as per the proof of concept demonstration here, Eve could then manipulate the mean photon number of the quantum states modulated by the QKD transmitter and bias the output of the QRNG in a controlled manner. If the device operator does not notice such an ongoing attack, the secret key / random number sequence from the QKD / QRNG device could be compromised. The manuscript is well written: equations are in an easy-to-read format, sectioning is logical, and photos, schematics, and graphs nicely illustrate the experiment. While the exploited vulnerability reported here is definitely novel and ingenious, the seriousness of its impact on the security of the system is somewhat debatable.

Based on experimental measurements conducted on (a fiber spool inside) the QKD transmitter and heuristics, the authors conclude that Eve would have to shine a few 100s of Watts of laser light to be able to inject a sufficient number of photons (on average) into the transmitter that then cause security violations. Would such a strong beam of light have absolutely no other influence? Would it not fry for instance the plastic sheath covering the fiber spool? Or badly injure a poor device operator who might accidentally walk into the beam path (The probability of a human operator walking into the beam path is probably a few orders of magnitude higher than the probability of Eve getting a LOS through ventilation holes to launch such an attack in the first place.) ? Or could multiple reflections and scatterings from such strong light beam result in some abnormal functioning of the device, thus disclosing the attack?

What I mean to say is, there are likely to be several unintended effects an actual attack may bring, and this has not been addressed at all. This group has in the past specialized in a variety of optical damage attacks so ideally speaking, they should perform some more measurements with the highest possible powers available and then include a frank discussion about telltales that Eve might leave behind. Also, given that the injection efficiency (free-space to fiber coupling via the reversible loss mechanisms) seems to be < -120 dB, it might make sense to compare the effectiveness of this attack with a traditional trojan attack where Eve shines light (through the quantum channel) at a wavelength long enough to prevent exciting the classical detectors photoelectrically. And they should definitely add a citation to a telecom wavelength source that produces 'realistically achievable c.w. power in the range of 200–500 W'.

Answer:

In order to address this and other comments, we have replaced c.w. power by the average energy an attacker needs to inject during the 20 ns the modulator is active. This formulation is more natural for pulsed lasers and helps to show the attack is realistic and less invasive than expected.
The figures in section III.D have been updated and we have expanded the discussion at the end of the section. We show the required energy ranges are achievable by many commercial lasers used in ranging (LIDAR) applications (we have added a list of suitable lasers). Depending on the exact repetition rate, the average power can actually be in the range of a few tens of W. This limits the possible damage to any operator or the surrounding equipment.

Some other questions and comments:

1. I guess the reason for using c.w. light for the demonstration here was to avoid the problem of synchronization of optical pulses to the gates of the single photon detector. But for an actual attack, isn't Eve somewhat limited by using c.w. light? As in, would a pulsed laser not be more practical?

Answer:

The experiment was thought to check whether it was possible or not to couple photons inside the fiber and c.w. was enough, but Eve would probably need a pulsed laser. While this adds a bit of complexity due to synchronization, the energy constraints relax. The end of Section III.D now discusses pulsed lasers and how they can help in the attack.

2. Section B, Page 8: "Comparing the coupling of light at 1310 and 1550 nm, we see that more photons enter the fiber at the lower 1310 nm wavelength, where Rayleigh scattering is stronger for silica fibers.

A natural question that arises here is: if more photons enter at 1310 nm, why is that not chosen as the attack wavelength? In general, what were the beam waists for these 2 wavelengths and how does the coupling vary if the beam waist is changed?

Answer:

Similarly to what happens for the 1550 experiment, the distance and the beam waist seem to be less important than the angle at which the light reaches the fiber spool. This measurement was made later on a similar spool of fiber to try to determine the main coupling mechanism. The beam waist in either measurement was much larger than the fiber diameter, i.e., the beam illuminated multiple fibers in the spool. We have now clarified this, in Sec. III B: "A fiber laser was connected to a fiber collimator a few centimeters from the ventilation hole giving a loosely focused spot on the fiber spool (of a couple millimeters diameter), illuminating multiple fibers in it."

In terms of an attack, the increase in the number of photons is modest but using a different wavelength adds a series of problems. Apart from making phase discrimination more difficult for the attacker (the PM works at 1550 nm), the attenuation of the phase modulator and the other elements in the way out would probably destroy any advantage. Note that Clavis2 has a narrowpass spectral filter at Bob's entrance, as part of a countermeasure against the (conventional) Trojan-horse attack.

3. Section B, Page 14: "We assume Eve knows the phase of the injected photons before the modulation and that she can establish their phase difference with respect to the reference pulses from Bob."

Might it not be simpler for Eve to discriminate the phase using homodyne detection, as was done in NJP 16, 123030 (2014)? (A phase reference can also be easily established since time periods with no modulation are equivalent to when a phase of 0 is modulated).

Answer:

The method to extract the phase was not clear in the original manuscript. We have modified the explanation and cited the eavesdropping experiment using homodyne detection as an example:

"Eve can compromise the system if she is able to deduce Alice's phase settings. If Eve uses pulses longer than the time bin the PM is active, each output photon is equivalent to the time bin qubits used in the QKD protocol. The part of the light reaching the inactive modulator serves as a phase reference. Experimentally, a simple alternative would be using a homodyne detection setup (see [57] for an example of the method in a Trojan-horse attack on QKD)."

4. Section B, Page 15: "Eve manages to inject a pulse with mean photon number μ inj towards the PM direction."

It would be nice to elaborate this sentence to avoid confusion. If I understood correctly, what is actually happening is that Eve shines a bright pulse which, due to the reversible loss coupling mechanism, results in a pulse with mean photon number μ inj that travels in the fiber towards the PM.

Answer:

The interpretation is correct. To explain it better, the sentence has been changed to:

"Let's assume that Eve sends external light that reaches the fiber spool and manages to couple a mean photon number μ_{inj} inside the fiber in a pulse towards the PM that passes the PM during its phase-modulation window."

5. Section C, Page 16: Was the fluctuation around the N = 60.8 value in the count rate negligible? If not, then mention that as well.

Answer:

We have included the standard deviation in the average injected photon number μ_{inj}:

"This gives the mean photon number per pulse (or per gate) $\mu_{\text{inj}} = 3.32(21) \times 10^{-3}$."

The Supplementary Data includes the count statistics for the experiments we have used to extrapolate the number of injected photons in our analysis and a description of the equipment we used. We assumed a Poisson statistic for the photon counts and the dark counts. The final standard deviation (of 0.21×10^{-3}) comes from propagating the uncertainty in the photon count and dark count statistics assuming the standard deviation is the square root of the mean (Poisson distribution).

We have replaced the mean values with those from a shorter experimental run for which we had the most reliable count statistics (due to a different counter used). The values and the results are similar to the ones we obtained with the originally listed data.

6. Section C, Equation 8: Add expression with an integer exponent to the RHS (e.g., $\mu_{ext} = 3.46 \times 10^{-6.3} = 1.73 \times 10^{-6}$) because otherwise it may cause a potential confusion when compared with μ_{inj}

Answer:

The equation has been modified to allow for an easier comparison:

$$\begin{aligned} \mu_{\text{ext}} &= 3.32 \times 10^{-6.3} = 1.56 \times 10^{-6}, && \text{(BB84)} \\ \mu_{\text{ext}} &= 3.32 \times 10^{-6.15} t^{-0.25} = 2.35 \times 10^{-6} t^{-0.25}. && \text{(SARG)} \end{aligned} \tag{1}$$

7. The caption in Fig. 6 reads "we take advantage of a ventilation hole at the top of the box". Is this is the beam splitter box? And it might make it easier for the reader to understand by adding the blue ray path in Fig. 6(b) as well.

Answer:

The ventilation hole is in the upper side of the case of the QRNG. We have modified Fig. 6 to show the path and the correspondence with the block diagram. We show the path to the pinhole of the beam splitter box.

B. Answers to reviewer 2:

In this article, the authors exploit ventilation holes in quantum devices to inject light inside it and either get information or modify the behaviour of the device itself.

In the first part of the article, the authors study a Trojan horse attack performed by injecting light directly into the SMF delay line of a commercial plug-and-play QKD system, thus preventing it from being detected by the photodiode placed at the entrance of the device. In the second part, they attack a simple QRNG prototype with the aim of inducing an imbalance in the detection rate of the two detectors, thus modifying the output statistics.

The idea behind the article is interesting, since it addresses some implementation issues that might completely break the security of the device. However, in my opinion, there are some major points, in the experimental evaluation of the attacks, that make the article, as it is now, unsuitable for publication.

To evaluate the effect of light injection on QKD, the authors shine a collimated laser into the ventilation holes of the Alice part of Clavis 2 QKD system, aiming at the fiber spool used as a delay line, and look at the number of photons just after the spool. By measuring the fraction of photons coupled into the fiber, they get the injection efficiency as a function of the input laser power and they estimate, from the losses of the different components in a typical QKD setting, the number of photons injected into the line going to Bob. An attacker can thus increase the number of photons going from Alice to Bob without being detected. The main problems in this part are:

** there is no description of the properties of the beam used in the injection. Since this article deals with attacks on the implementation, it should contain more information on how this attack can be implemented practically.*

Answer:

Section III has been extended to discuss the feasibility of light injection with existing commercial lasers. It also includes a description of the setup we used. In our experiment, we used a fiber laser connected to a fiber collimator at a short distance (cm) from the ventilation hole and beam divergence was not critical. However, in the discussion about existing lasers which are potentially useful for a ventilation attack, we notice that most of them are used in ranging applications and have a beam quality factor close to the diffraction limit allowing attacks from a distance. Section III B now discusses the beam and timing parameters of the laser and Sec. III D studies the range of feasible powers for an attacker who has access to a pulsed laser.

** the analysis of the attack considers just the effect of an undetected increase of the mean photon number coming out from Alice. What would change if Eve used a light at a slightly different wavelength, putting a wave-division multiplexer in the channel collecting all the light she injected without affecting the one going to Bob?*

Answer:

This possibility was already mentioned in the text:

"If Eve injects photons at a slightly different wavelength than the photons from Bob, she could also tell apart the injected photons from the rest with a wavelength demultiplexer, if needed."

However, given the low coupling we found in our device, Eve's best strategy seems to be using also the photons in the legitimate channel. If the coupling were better, using a slightly different wavelength could result in a simpler attack.

** the authors base the analysis on the attack model described in ref [57]. One of the hypothesis in their analysis is that Eve measures her quantum system just after the sifting procedure. This requires her to have a quantum memory, something that is not realistic nowadays.*

Answer:

While this is correct, we believe a QKD system should be designed to be as close to ideal as possible and be resistant to current and future attacks. For this example we could only couple a few photons, but, as hidden light paths are not actively considered during the design of quantum devices, there might be configurations for which the coupling is higher and there exist simple attacks (like the QRNG example).

Furthermore, the point of using QKD is giving a protection derived from the laws of quantum mechanics. From the moment there is a gap between the expected and the real behaviour, the protocol can no longer promise this security. We have now made this point in the text:

"While the known combined photon-number-splitting and cloning attacks [58] require a quantum memory, the difference between the assumed and the actual key rates opens a loophole in the security of the system and must be fixed. Otherwise we cannot claim physical security where the only limitation on the attacker is what is allowed by the laws of quantum mechanics. A QKD system should be safe not only against present attacks, but also against future, more technologically advanced attackers (who may have a quantum memory)."

Given that the countermeasures are simple and low in cost (covering the optical parts), the main purpose of the paper is to advocate for a careful design by showing that an attack is, in principle, possible but easy to avoid with a little forethought.

** the authors show their attack to be effective for laser powers going from 200 to 500 W. While it is true that these powers are realistically achievable, it is not clear that they don't give rise to other issues (like the degradation of the components). I think the authors should be more careful about this point, since it is crucial in their work.*

Answer:

This comment has led to our main revision in the paper. Section III B and III D now explain how an attacker can use pulsed lasers to inject the required number of photons while using a relatively low average power in the order of a few watt to tens of watt.

We have modified the discussion to talk in terms of the pulse energy that needs to be achieved in each 20 ns data pulse, which is more natural for pulsed lasers than speaking of c.w. power. Figures 4 and 5 have been updated accordingly. We show that the pulse energies required for an attack can be achieved by many commercial lasers used in ranging (LIDAR) applications. We have added a list of potentially suitable lasers. With pulsed lasers, the average power is actually in the range of a few watt, making damage to the surrounding components less likely.

In practice, light does not have a single point of insertion to the fiber and the range of powers for the attack will be in between the c.w. limit and the pulsed laser limit. This has been discussed in Secs. III B and III D.

The following section, about the attacks through ventilation holes on a QRNG, is even more problematic. Indeed, the authors just say that injecting light into the QRNG it is possible to bias the statistics of the two detectors, something that is well known. The prototype QRNG they show in the photo is almost 20 years old, so it has nothing to do with current technology in QRNGs. Moreover, there are no data supporting the claims made by the authors (except a generic "we managed to obtain ratio > 80% ...", with no description of the setup used for obtaining it). In order to get something meaningful, the authors should at least compare the statistics obtained with and without the attack, showing explicitly which advantage could an adversary gain with it.

Answer:

We believe that, even though the attack is simple, it underscores our point that any optical quantum system should be designed with light paths in mind. While it can be argued that it is obvious that any additional light will bias the output, this is, to our notice, the first example of an attack using a hidden light path to alter the output of a QRNG. In general, it seems light injection attacks are not considered during design.

The results do not generalize well to QRNGs with integrated optics, but could be extended to "bulkier" devices. In particular, the prototype, while not a commercial device, has evolved since the original paper and different units are routinely used in experiments and they have their own control software, making them a good platform to study optical QRNGs. We have added an explanation in Sec. IV:

"Three units of the prototype were used in research setups. We did most of our work on two of them and obtained similar results. There are small variations between the units and they have their own control software, very similar to what can be expected from a commercial device."

Regarding the details of the attack, we have expanded the description and now include explicit details on how the resulting bit sequences are biased after the attack:

"Our attack works by flooding the legitimate photons from the LED with a much stronger beam coming from the outside. We produced the latter with a handheld LED flashlight (Mini-Maglite AA). The injected light reaches the pinhole and, once inside the box and depending on the angle, it is reflected and scattered in multiple directions. By manually varying the input angle, we could maximize the amount of light going into PMT2. The increased photon number in just one detector biases the sequence in favor of 1s in the device we did most of the work with (in other units of the prototype we found the detectors associated with 1 and 0 were swapped).
This rudimentary attack is enough to show that it is possible to bias the output sequence. We used the control software to store two sequences of around 15 MB, one generated under normal operation and one generated while directing the output of the flashlight to the ventilation hole. We managed to obtain a ratio $> 80\%$ for the number of ones in the final generated sequence just by moving the flashlight into an adequate angle (99200493 bits were 1 while 22503955 bits were 0). Using the Linux utilities `ent` [67] and `rngtest` [68] we could also see that, while under normal operation the results of the χ^2-test [69] and the FIPS-140-2 tests [46] were consistent with a uniform random sequence, the bits generated during the light injection attack failed these tests. The sample files are included in the Supporting Information, together with the results of applying the different randomness tests to the sequences."

The most important change is in the Supporting Information, which now includes two files with random sequences generated during normal operation and with light from a LED flashlight directed towards the ventilation hole. Apart from the binary files that allow to reproduce the ratio between 0s and 1s, we have included the results from two randomness tests (using the utilities `ent` and `rngtest`) that show that under normal operation the bits perform well under the χ^2-test or the FIPS 140-2 tests but the output sequences are heavily biased under the attack.

Finally we have added an example on how biased random numbers can compromise security with a new reference to the effects of imperfect randomness in QKD:

"This is more than enough to launch a successful attack and reduce the entropy of the output in this particular QRNG design. Even small biases in the random sequences can compromise many cryptographic protocols [70]. For instance, in QKD, if the random numbers in the basis and bit selection are biased, the protocol becomes insecure [71].
A light injection attack is not limited to biasing the bit sequence towards one of the values. At the beginning of the random bit generation, there is a calibration stage during which the PMT voltages are adjusted to optimize detection [66]. An attacker could introduce an intermediate level of light for the calibrated state (50% generation rate for 0s and 1s). By removing the light Eve can increase the probability of getting 0. By increasing the light, she can bias the bit towards 1. The light could be invisible for the people in the room. While we used white light, the detectors' efficiency peaks at UV wavelengths and an attacker could hide the injected light from the operators of the QRNG."

As a summary, the authors address a real security issue in the security of quantum devices, i.e., the accessibility of optical components through ventilation holes, but they don't provide enough evidence on how this could be exploited, now, by an eavesdropper. I think that, before being considered again for publication, the authors should address more carefully all the experimental aspects behind the proposed attacks, focusing on what is feasible with current technology.

Answer:

With the changes in Sec. III and the more detailed description of how the light affects the output of the QRNG, we hope we have clarified what is possible with the current technology. In any case, given that the solution is simple and inexpensive, we believe it is worth protecting quantum systems against any external light even if an attack is deemed to be too sophisticated or unlikely, similarly to what is recommended in standards of classical cryptographical devices with regard to tampering through ventilation holes (see Sec. V).
